# Antioxidant and Angiotensin-Converting Enzyme Inhibitory Activity of Faba Bean-Derived Peptides After *In Vitro* Gastrointestinal Digestion: Insight into Their Mechanism of Action

**DOI:** 10.1021/acs.jafc.4c00829

**Published:** 2024-03-12

**Authors:** Delphine Martineau-Côté, Allaoua Achouri, Salwa Karboune, Lamia L’Hocine

**Affiliations:** † Agriculture and Agri-Food Canada, Saint-Hyacinthe Research and Development Centre, Saint-Hyacinthe, Quebec J2S 8E3, Canada; ‡ Department of Food Science and Agricultural Chemistry, Macdonald Campus, McGill University, Sainte-Anne-de-Bellevue, Quebec H9X 3 V9, Canada

**Keywords:** faba bean, *Vicia faba L.*, pulse protein, antioxidant, ACE inhibitor, multifunctional peptides, synergism, molecular docking

## Abstract

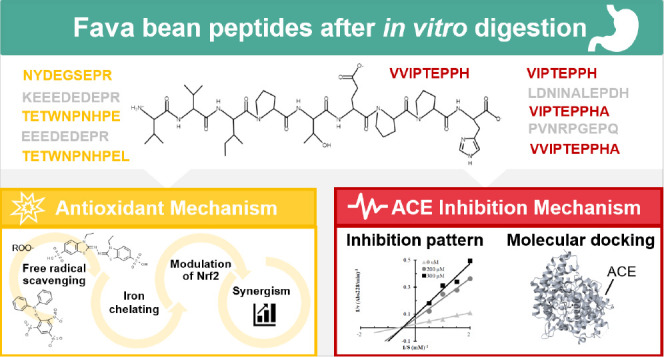

Faba bean flour, after *in vitro* gastrointestinal digestion, showed important antioxidant and angiotensin-converting enzyme (ACE) inhibitory activities. In the present study, 11 faba bean- derived peptides were synthesized to confirm their bioactivities and provide a deeper understanding of their mechanisms of action. The results revealed that 7 peptides were potent antioxidants, namely, NYDEGSEPR, TETWNPNHPEL, TETWNPNHPE, VIPTEPPH, VIPTEPPHA, VVIPTEPPHA, and VVIPTEPPH. Among them, TETWNPNHPEL had the highest activity in the ABTS (EC_50_ = 0.5 ± 0.2 mM) and DPPH (EC_50_ = 2.1 ± 0.1 mM) assays (*p* < 0.05), whereas TETWNPNHPE had the highest activity (*p* < 0.05) in the ORAC assay (2.84 ± 0.08 mM Trolox equivalent/mM). Synergistic and/or additive effects were found when selected peptides (TETWNPNHPEL, NYDEGSEPR, and VVIPTEPPHA) were combined. Four peptides were potent ACE inhibitors, where VVIPTEPPH (IC_50_ = 43 ± 1 μM) and VVIPTEPPHA (IC_50_ = 50 ± 5 μM) had the highest activity (*p* < 0.05), followed by VIPTEPPH (IC_50_ = 90 ± 10 μM) and then VIPTEPPHA (IC_50_ = 123 ± 5 μM) (*p* < 0.05). These peptides were noncompetitive inhibitors, as supported by kinetic studies and a molecular docking investigation. This study demonstrated that peptides derived from faba beans have multifunctional bioactivities, making them a promising food-functional and nutraceutical ingredient.

## 1 Introduction

According to the World Health Organization (WHO), noncommunicable diseases are the world-leading cause of premature death, with cardiovascular diseases, being the leading cause of mortality, followed by cancer and respiratory disease.^1^ Hypertension is a well-recognized risk factor for cardiovascular diseases^2^ that can be managed through medication and a healthy lifestyle.

An important pharmacological target for the treatment of hypertension is the angiotensin-converting enzyme (ACE). ACE is a zinc dipeptidyl carboxypeptidase that plays a critical role in the regulation of blood pressure and cardiovascular function through the renin–angiotensin–aldosterone system (RAAS)^3^ and the kallikrein–kinin system (KKS).^4^ In the RAAS, ACE hydrolyzes angiotensin I to form the potent vasoconstrictor angiotensin II, while in the KKS, ACE converts bradykinin, a potent vasodilator to an inactive fragment.^4^ Numerous synthetic ACE inhibitors, such as captopril and linosipril, among others, have been used for decades to treat hypertension.

In addition to RAAS deregulation, increased oxidative stress has also been linked to the development of hypertension and noncommunicable diseases.^5^ Oxidative stress occurs when there is an imbalance between the generation of reactive oxygen species (ROS) and the antioxidant defense system, leading to an imbalance of the redox cellular signaling pathways, and thus, molecular damages.^6^ Oxidative stress can result in endothelial and renal damage, vascular dysfunction, and cardiovascular fibrosis, all of which are known to play a role in the development of hypertension.^6^ The new generation of drugs with dual ACE inhibition and antioxidant effects are, therefore, regarded as promising alternatives to treat high blood pressure and prevent cardiovascular diseases.^7,8^

Concomitant with medication, a healthy lifestyle can have a protective effect against hypertension.^9^ In this vein, recent reports have suggested that pulse consumption is associated with a blood pressure-lowering effect.^10−12^ Several pulse components, such as phenolic compounds, γ-aminobutyric acid (GABA), and dietary fibers, are believed to contribute to this hypotensive effect.^13^ The release of antioxidant and ACE inhibitor peptides after gastrointestinal digestion of pulse proteins also has the potential to contribute to this beneficial heath effect.

Faba bean (*Vicia faba* L.) is an emerging high-quality and sustainable pulse protein source with promising health benefits.^14^ In a previous work, we demonstrated that faba bean flours after *in vitro* gastrointestinal digestion^15^ have a high antioxidant and ACE inhibitory effect, which could play a role in hypertension management.^16^ The faba bean peptides present in the *in vitro* gastrointestinal digestate complex mixture^15^ were enriched through a 3 kDa cutoff membrane ultrafiltration followed by preparative size exclusion chromatography and sequenced by mass spectrometry. The obtained peptide-enriched fractions maintained a high antioxidant and ACE inhibition effect, demonstrating that these health-beneficial bioactivities were related to peptides.^16^

The objective of the present work was to further ascertain the antioxidant and ACE inhibitory activities of faba bean derived-peptides and gain a new understanding of their mode of action. To this end, the highly active faba bean peptides^16^ were chemically synthesized (Table 1) and tested individually for antioxidant and ACE inhibition activity. The mechanisms of action of antioxidant peptides were investigated using a combination of *in vitro* and cellular antioxidant assays. The mechanisms of action of ACE inhibitor peptides were investigated through enzyme kinetic studies to determine the inhibition pattern and molecular docking to assess and compare their potential binding mode to ACE. This is the first study reporting an in-depth investigation of the mechanisms of action of faba bean-derived bioactive peptides after gastrointestinal digestion with multifunctional and synergistic activities, using a combination of *in silico*, *in vitro*, and cellular models.

**Table 1:** List of Synthesized Peptides Identified from Faba Bean Flour *In Vitro* Gastrointestinal Digestate^16^

peptides	parent protein	protein accession number	fragment location	% hydrophobic residue^a^
NYDEGSEPR	convicilin	B0BCL8	29–37	11.11
PVNRPGEPQ	vicilin	I0B569	152–160	44.44
LDNINALEPDH	legumin B	P05190.1	35–45	45.45
TETWNPNHPEL			52–61	36.36
TETWNPNHPE			52–62	30.00
EEEDEDEPR	legumin	Q43673	327–335	11.11
KEEEDEDEPR			326–335	10.00
VIPTEPPH	tonoplast intrinsic protein 32	A0A024NRI7	155–162	62.50
VIPTEPPHA			155–163	66.67
VVIPTEPPHA			154–163	70.00
VVIPTEPPH			154–162	66.67

a Hydrophobic and uncharged residue are phenylalanine (F), isoleucine (I), leucine (L), methionine (M), valine (V), tryptophan (W), alanine (A), and proline (P).

## 2 Materials and Methods

### 2.1 Chemicals

The peptides derived from faba bean flour gastrointestinal digestate NYDEGSEPR, PVNRPGEPQ, LDNINALEPDH, TETWNPNHPEL, TETWNPNHPE, EEEDEDEPR, KEEEDEDEPR, VIPTEPPH, VIPTEPPHA, VVIPTEPPHA, and VVIPTEPPH (Table 1) were synthesized by Biomatik (Kitchener, Ontario, Canada). Their purity (>98%) and quality were checked by reverse-phase HPLC (>98%) and mass spectrometry analysis.

Angiotensin-converting enzyme (ACE) from rabbit lung (A6778), N-hippuryl–His–Leu hydrate (HHL) (H1635), and captopril were purchased from Sigma-Aldrich (St. Louis, MO, USA).

For cell culture, minimum essential medium (MEM), nonessential amino acid solution 100 × , heat-inactivated fetal bovine serum (FBS), 5000 IU penicillin, and 5000 μg/mL streptomycin solution were purchased from Wisent Bioproducts (Saint-Jean-Baptiste, QC, Canada). Sodium pyruvate (100 mM) was purchased from Cytiva (Uppsala, Sweden). Geneticin (50 mg/mL) was purchased from Gibco (Thermo Fisher Scientific, San Jose, CA, USA). Antioxidant response element (ARE) reporter–HepG2 cells and the One-Step Luciferase Assay System were purchased from BPS Bioscience (San Diego, CA, USA). All chemicals and reagents used were of analytical grade. Deionized water was used in all of the experiments.

### 2.2 Antioxidant Mechanism of Faba Bean-Derived Peptides

#### 2.2.1 In Vitro Antioxidant Assays

2,2-Diphenyl-1-picrylhydrazyl (DPPH), 2,2′-azinobis(3-ethylbenzothiazoline-6-sulfonic acid) (ABTS), Oxygen Radical Absorption Capacity (ORAC), and the iron chelating assays were performed following the methods of Orona-Tamayo, Valverde, Nieto-Rendón, and Paredes-López (2015),^17^ Re, Pellegrini, Proteggente, Pannala, Yang, and Rice-Evans (1999),^18^ Tomer, McLeman, Ohmine, Scherer, Murray, and O’Neill (2007),^19^ and of Orona-Tamayo et al. (2015),^17^ respectively, as described in Martineau-Côté, Achouri, Wanasundara et al. (2022).^16^ For the DPPH, ABTS, and iron chelating assays, the peptides were first screened at a high dose (10 mM). Peptides with antioxidant activity were then tested at different concentrations (0.1–10 mM) to evaluate the dose–response effect. The results were expressed as the half-maximal effective concentration (EC_50_), which was defined as the required peptide concentration, leading to 50% scavenging or chelating activity. The EC_50_ values were calculated using a four-parameter logistic curve regression, and Trolox was used as a positive control. For the ORAC assay, the results were expressed as μmol of Trolox equivalent per mM of peptides.

#### 2.2.2 Investigation of Potential Additive, Synergistic, And/Or Antagonist Interactions of Faba Bean-Derived Antioxidant Peptides

Since the synthesized faba bean-derived peptides had individually lower antioxidant activities than the complete faba bean flour digestate with the DPPH and ABTS assays, we investigated whether some peptide combinations were additive or synergistic. To this end, the method of Chou and Talalay (1984)^20^ was used to determine the combination index (CI) and the dose reduction index (DRI) of selected peptides combinations. The peptides were tested individually and in combination using a constant ratio (i.e., the ratio of their EC_50_) at various concentrations. The DPPH and ABTS assays were performed as described in Section 2.2.1.

Data analysis was performed using the CompuSyn software^21^ (ComboSyn Inc.) to calculate the CI and the DRI values at various levels of free radical scavenging activity (Fa). The CI values were used to determine the type of interaction between peptides, where CI < 1, CI = 1, and CI> 1 indicate synergism, additivity, and antagonism, respectively. DRI represents the dose reduction fold that can be achieved for a given peptide when used in combination. DRI values above 1 indicate that dose reduction is favorable, whereas a value below 1 indicates that dose reduction is unfavorable.

#### 2.2.3 Modulation of the Nuclear Factor Erythroid 2–related Factor 2 (Nrf2-ARE) Cellular Pathway by Faba Bean-Derived Antioxidant Peptides

The most potent antioxidant peptides identified with *in vitro* antioxidant assays were tested at the cellular level using the Nrf2-ARE live cell assay as described by Vigliante, Mannino, and Maffei (2019)^22^ with minor modifications. The HepG2 cells transfected with a firefly luciferase gene under the control of ARE were routinely cultivated in growth medium, which was composed of MEM supplemented with 10% FBS, 1% nonessential amino acids, 1 mM sodium pyruvate, 1% penicillin and streptomycin solution, and 600 μg/mL Geneticin at 37 °C in an atmosphere containing 5% CO_2_. Cells were subcultivated at 90% confluence using a split ratio of 1:5. Cells between passages 3 and 11 were used in the experiments.

The activation of the Nrf2-ARE pathway by faba bean-derived peptides was investigated both in basal conditions and in the presence of oxidative stress (H_2_O_2_ 0.25 mM). To this end, 4 × 10^4^ cells in 45 μL of growth medium without Geneticin were added to 96-well white microplates with clear bottoms. Five μL of faba bean-derived peptides were added in triplicate to reach a final concentration of 1, 0.5, or 0.05 mM, with or without 0.25 mM H_2_O_2._ Tert-butylhydroquinone (TBHQ) was used as a positive control, and assay medium with and without 0.25 mM H_2_O_2_ was used as negative controls. The plates were incubated for 18 h at 37 °C in an atmosphere containing 5% CO_2_.

The next day, the activation of the Nrf2-ARE pathway was quantified using the One-Step Luciferase Assay System (BPS Bioscience, San Diego, CA, USA) as described by the manufacturer. Briefly, 100 μL of the luciferase assay working solution equilibrated at room temperature was added to each well. The plate was incubated for 15 min at room temperature with constant stirring. Luminescence was recorded with a Synergy HTX microplate reader (Bio-Tek, Winooski, VT, USA). ARE modulation was expressed as a fold increase compared to the negative control using the following formula after background subtraction:
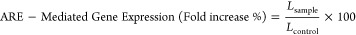
1

where *L*_sample_ is the relative luminescence reading of the cells treated with the faba bean-derived peptides and *L*_control_ is the relative luminescence reading of untreated cells.

### 2.3 ACE Inhibition Mechanism of Faba Bean-Derived Peptides

#### 2.3.1 *In Vitro* ACE Inhibitory Activity

ACE inhibition activity was measured following the protocol of Barbana and Boye (2011)^23^ as described in Martineau-Côté, Achouri, Wanasundara et al. (2022).^16^ ACE from rabbits was used for *in vitro* testing, since rabbit and human ACE are nearly homologous and their active sites are highly similar.^24^

#### 2.3.2 Determination of ACE Inhibition Pattern

A kinetic study was performed following the procedure of Barbana et al. (2011)^23^ to determine the inhibition pattern of four faba bean peptides with ACE inhibition activity (VIPTEPPH, VIPTEPPHA, VVIPTEPPHA, and VVIPTEPPH). The initial rate of reaction was measured with different HHL (0.5–2 mM) and peptides concentrations. Lineweaver–Burk double reciprocal plots were built to identify the inhibition pattern.

#### 2.3.3 Elucidation of the Peptide Binding Mode by Molecular Docking

Molecular docking was used to identify the potential binding mode of faba bean-derived peptides (VIPTEPPH, VIPTEPPHA, VVIPTEPPHA, and VVIPTEPPH) to ACE. The peptide structures were created using PEP-FOLD 3.5.^25^ The crystal structure of the C-domain of somatic human ACE (PDB: 4APH, resolution: 1.99 Å) was retrieved from the RCSB protein databank (https://www.rcsb.org). The PDB file was edited to remove any molecules except the protein chain, zinc ion, two chlorine ions, and angiotensin II. Angiotensin II was kept in the docking simulation since the kinetic study revealed that the four faba bean peptides were noncompetitive inhibitors, meaning that they can bind ACE whether or not the substrate is binding the active site. Angiotensin II was used to simulate the enzyme substrate since no crystal structure of Angiotensin I with ACE is available.

The most probable binding sites between ACE and the four peptides were predicted using HPEPDOCK,^26^ a global flexible peptide protein docking software. Global docking enables a blind docking simulation on the whole protein chain when the binding site is unknown. The most probable model for each peptide was selected based on the lowest HPEPDOCK docking score. The ACE and faba bean-derived peptide complexes were further analyzed with Ligplot+^27^ to identify molecular interactions. Molecular graphics were produced with UCSF ChimeraX.^28^

The peptide protein docking procedure was validated with two controls: angiotensin II and the bradykinin-potentiating peptide b (BPPb). Angiotensin II and BPPb were extracted from their cocrystallized structure with ACE (PDB 4APH and 4APJ, respectively) and redocked with ACE. The docked poses were compared to the crystal structure through root-mean-square deviation (RMSD) and comparison of the molecular interaction stabilizing the complexes.

### 2.4 Statistical Analysis

Each analysis was performed in triplicate, and the results were expressed as the mean ± standard deviation (SD). The data were analyzed through analysis of variance (ANOVA) (*p* < 0.05) followed by the Tukey’s honest significant difference (HSD) posthoc test (*p* < 0.05) or the Dunnett’s posthoc test, using the XLSTAT software (Addinsoft, NY, USA) add-on to Microsoft Excel (Redmond, WA, USA) to determine significant differences.

## 3 Results and Discussion

### 3.1 Antioxidant Mechanism of Faba Bean-Derived Peptides

#### 3.1.1 In Vitro Antioxidant Activity of Faba Bean-Derived Peptides

**Figure 1:**
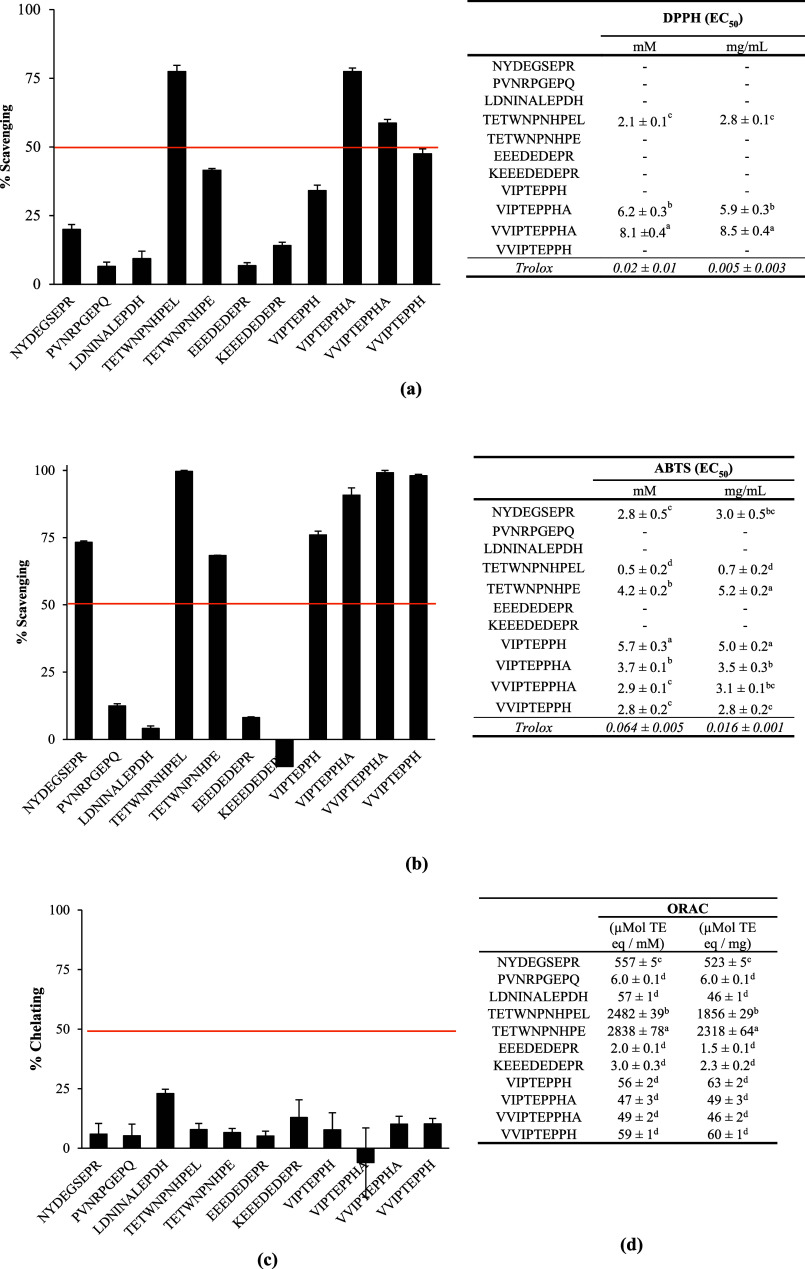
Antioxidant activity (mean ± standard deviation) of faba bean-derived peptides was assayed with (a) the DPPH, (b) the ABTS, (c) the iron chelating, and (d) the ORAC assay. For the DPPH, the ABTS, and the iron chelating assay, a first screening was performed at a high peptide concentration (10 mM) and the EC_50_ was determined for the peptides with an activity. For the ORAC assay, the data were expressed as μMol of Trolox equivalent. Means without a common letter differ (*p* < 0.05), as analyzed by one-way ANOVA and Tukey’s test.

The 11 faba bean-derived peptides (Table 1) were first tested for *in vitro* antioxidant activity (Figure 1). Among them, 7 peptides were potent free radical scavengers when assessed with 2,2-diphenyl-1-picrylhydrazyl (DPPH), 2,2-azinobis (3-ethylbenzothiazoline-6-sulfonic acid) ABTS, and/or Oxygen Radical Absorption Capacity (ORAC) assay (Figure 1), namely, TETWNPNHPEL, TETWNPNHPE, NYDEGSEPR, VIPTEPPH, VIPTEPPHA, VVIPTEPPHA, and VVIPTEPPH. These 7 peptides had in their primary sequence an amino acid recognized for free radical scavenging activity, such as tryptophan (W), tyrosine (Y), and/or histidine (H),^29^ which may partly explain these antioxidant properties.

The peptides TETWNPNHPEL and TETWNPNHPE had a very high activity in the ORAC assay, which was 2.5 to 2.8 times higher than Trolox on a molar basis (Figure 1). The presence of tryptophan (W) and histidine (H) in these peptide sequences is likely to contribute to this free radical scavenging property. The activities of TETWNPNHPEL and TETWNPNHPE, when expressed in μMol of Trolox equivalent per mg of peptides (1.9 and 2.3 μMol Trolox eq/mg, respectively), were higher than the activity measured in the faba bean peptide-enriched fraction (0.7 μMol Trolox eq/mg) and slightly lower than the activity measured in the complete 3 kDa permeate of faba bean digestate (2.7 μMol Trolox eq/mg). This finding means that TETWNPNHPEL and TETWNPNHPE are very important contributors of this activity in the complete 3 kDa permeate of faba bean digestate. The peptide NYDEGSEPR also had a high activity in the ORAC assay, which was equivalent to half that of Trolox on a molar basis. The dipeptide NY, present at the N-terminal extremity of NYDEGSEPR, was shown to have a strong free radical scavenging activity in the ORAC assay (3246 μMol TE eq/mM) and in the ABTS assay (EC_50_ = 8.3 μM).^30^ This peptide fragment is, therefore, undoubtedly an important contributor to the activity of NYDEGSEPR. The significantly higher activity of TETWNPNHPEL and TETWNPNHPE compared to NYDEGSEPR, VIPTEPPH, VIPTEPPHA, VVIPTEPPHA, and VVIPTEPPH (Figure 1) (*p* < 0.05) could be explained by their respective amino acid composition, since tryptophan (W) was shown to have a higher free radical scavenging activity in the ORAC assay (2790 μMol TE eq/mM) compared to tyrosine (Y) (1020 μMol TE eq/mM) and histidine (78 μMol TE eq/mM).^31^

None of the identified faba bean peptides were potent iron chelators (Figure 1). This finding was surprising since a high-chelating activity was measured in the 3 kDa permeate of faba bean flour digestate. The synthesized peptides were tested at a high concentration (10 mM), corresponding to ∼9,000 to ∼13,000 μg/mL. These concentrations are superior to the EC_50_ of the digestate permeate (146 μg/mL),^16^ indicating that the iron chelating activity of the permeate might be explained by the contribution of smaller peptides that were not detected or other bioactive components, such as polyphenols.

Seven peptides showed potent antioxidant activity with the ABTS assay (NYDEGSEPR, TETWNPNHPEL, TETWNPNHPE, VIPTEPPH, VIPTEPPHA, VVIPTEPPHA, and VVIPTEPPH) and among these, three exhibited high activity with the DPPH assay (TETWNPNHPEL, VIPTEPPHA, and VVIPTEPPHA) (Figure 1). TETWNPNHPEL was the most potent antioxidant peptide in both assays. Interestingly, the EC_50_ (mg/mL) values of these 7 peptides were higher than those of the 3 kDa permeate of faba bean flour digestate. The EC_50_ of the individual peptides ranged from 0.7 to 5.2 and 2.8 to 8.5 mg/mL, compared to 0.1 and 0.8 mg/mL for the 3 kDa permeate of faba bean flour digestate in the ABTS and DPPH assays, respectively. Therefore, the activities of the individual peptides were at least 4 times lower than the complete 3 kDa permeate of faba bean digestate. This finding means that the activity measured in the complete permeate digestate is likely the result of an additive or synergistic effect of a combination of these peptides. Moreover, the contribution of other bioactive constituents of the faba bean matrix in the permeate digestate, such as polyphenols and oligosaccharides, cannot be excluded. Indeed, the crude characterization of the faba bean digestate 3 kDa permeate was performed in a previous study,^16^ and in addition to peptides (34.4 g/100 g), it contained 4.8 mg/g of total polyphenols (expressed as gallic acid equivalent) and 43.5 g/100 g of total carbohydrates (expressed as glucose equivalent).

Since the activities of TETWNPNHPEL and TETWNPNHPE were 2.5 to 2.8 times higher than Trolox in the ORAC assay but 8 to 66 times lower than Trolox in the ABTS assay, it can be hypothesized that their mechanism of free radical scavenging is essentially based on hydrogen atom transfer (HAT) rather than single electron transfer (SET). Similar results were obtained for VIPTEPPH, VIPTEPPHA, VVIPTEPPHA, and VVIPTEPPH, whose activities were 17 to 21 times lower than Trolox in the ORAC assay, compared to 44 to 89 times lower in the ABTS assay, favoring a HAT-based mechanism.

Interestingly, the results showed that minor modifications of the amino acid sequence led to important variations in the antioxidant potency of faba bean-derived peptides in the ABTS and DPPH assays (Figure 1). For instance, the leucine residue at the C-terminal position of TETWNPNHPE**L** seems to be crucial in the antioxidant activity, since its removal resulted in a significant increase (*p* < 0.05) of the EC_50_ by a factor of 8.4 in the ABTS assay and a loss of activity in the DPPH assay. This finding is in good agreement with a previous report, where the fragments EL and PEL demonstrated strong free radical scavenging activity in the DPPH assay, when present at the C-terminal extremity of a casein-derived peptide.^32^ Moreover, this leucine residue at the C-terminal extremity increases the percentage of hydrophobic residue (Table 1), which is known to favorably affect the antioxidant activity.^33,34^ Similarly, the alanine residue at the C-terminal extremity of VIPTEPPH**A** and VVIPTEPPH**A** was revealed to be essential in the DPPH assay, since its removal caused a decrease in activity in VIPTEPPH and VVIPTEPPH, respectively. This could be attributed to the fact that the fragment PHA has demonstrated strong antioxidant activity.^35^ Moreover, the additional valine residue at the N-terminal extremity of **V**VIPTEPPHA and **V**VIPTEPPH caused a significant increase of the antioxidant potency in the ABTS assay compared to VIPTEPPHA and VIPTEPPH, respectively (*p* < 0.05). This is in good agreement with previous reports, indicating that the presence of hydrophobic amino acids at the N-terminal extremity of peptides, such as valine, alanine, leucine, and isoleucine, is an important contributor to free radical scavenging properties.^34,36^ Moreover, the additional valine at the N-terminal extremity of **V**VIPTEPPHA and **V**VIPTEPPH increased the % of hydrophobic residue in the peptide sequence compared to VIPTEPPHA and VIPTEPPH, respectively (Table 1), further supporting the importance of the hydrophobic residue in the antioxidant activity. In the same vein, it is noted that VIPTEPPH had the lowest free radical scavenging activity in the ABTS and DPPH assays, coinciding with the lowest percentage of hydrophobic residue in its sequence compared to VVIPTEPPHA, VVIPTEPPH, and VIPTEPPHA.

#### 3.1.2 Investigation of Potential Additive, Synergistic, And/Or Antagonist Effects of Selected Faba Bean-Derived Antioxidant Peptide Combinations

**Figure 2:**
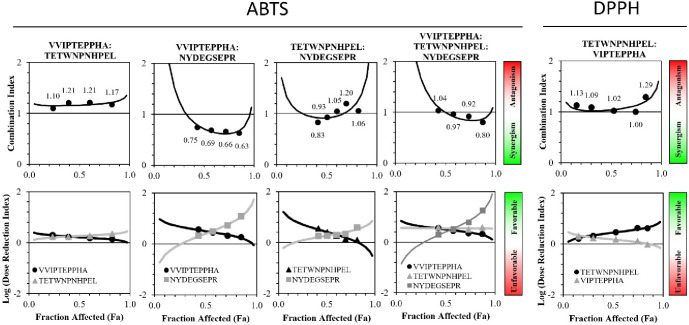
Combination index (CI) and dose reduction index (DRI) at different levels of free radical scavenging activity (F_a_) of faba bean-derived peptide combinations; (a) ABTS assay; (b) DPPH assay; the CI and the DRI were calculated based on the method of Chou et al. (1984)^20^ with the CompuSyn software.^21^ The dots represent the experimental data, and the lines are the fitted data. Synergistic effects are defined as CI < 1, additive effects are CI = 1, and antagonistic effects are CI > 1.

Since the synthesized peptides had individually lower antioxidant activities in the ABTS and DPPH assays than the complete 3 kDa permeate of faba bean *in vitro* gastrointestinal digestate, we investigated whether some of these peptides were having additive or synergistic effects using the Chou et al. (1984)^20^ method. Several studies have reported a lower antioxidant activity of peptide- enriched fractions and/or synthesized peptides compared to the complete protein hydrolyzate, suggesting synergistic interactions between the different peptides.^37−39^ Nonetheless, very few studies have investigated the synergistic and antagonistic interactions of specific peptide combinations to gain a better insight into this phenomenon.^38,40^ The mechanisms behind such interactions between peptides are widely unknown. Combinations of the most potent faba bean- derived antioxidant peptides with different amino acid chains were tested. The selected combinations for the ABTS assay were VVIPTEPPHA and TETWNPNHPEL, VVIPTEPPHA and NYDEGSEPR, TETWNPNHPEL and NYDEGSEPR, and finally VVIPTEPPHA, TETWNPNHPEL, and NYDEGSEPR. For the DPPH, the combination of VIPTEPPHA and TETWNPNHPEL was tested (Figure 2).

The combination index plot for each peptide combination was generated to identify synergistic, additive, and/or antagonist interactions (Figure 2) at different levels of free radical scavenging activity. A CI value <1 indicates synergism, CI = 1 indicates additivity, and CI > 1 indicates antagonism. The level of synergism and antagonism can also be evaluated based on the CI value, as a CI value <0.1 shows very strong synergism, 0.1–0.3 strong synergism, 0.3–0.7 synergism, 0.7–0.85 moderate synergism, 0.85–0.90 slight synergism, 0.9–1.1 additive, 1.1–1.2 slight antagonism, 1.2–1.45 moderate antagonism, 1.45–3.3 antagonism, 3.3–10 strong antagonism, and >10 very strong antagonism.^41^

For the combination of VVIPTEPPHA and TETWNPNHPEL in the ABTS assay, the CI values of the four data points ranged from 1.10 to 1.21, showing an additive to a slight antagonist interaction. As these two peptides have a high proportion of hydrophobic residue (Table 1), it can be hypothesized that once combined, hydrophobic interactions are formed, decreasing the availability of tryptophan and histidine to scavenge the ABTS radical. On the contrary, the combination of VVIPTEPPHA and NYDEGSEPR was synergistic for the four data points. The peptide NYDEGSEPR is more hydrophilic than VVIPTEPPHA (Table 1), which may decrease peptide interactions. The combination of TETWNPNHPEL and NYDEGSEPR was additive for 3 data points, moderately synergistic for one point, and slightly antagonistic for one point, showing that the type of interaction is dependent on the level of free radical scavenging activity. Since the interaction was mainly additive, it means that the activities of the two peptides are independent, indicating that both reacted with the ABTS radical in a similar manner and that there are limited interactions between the two peptides. This indicates that both peptides react with the ABTS radical in a similar manner and that there are limited interactions between the two peptides. Contrarily to our results, Jia, Zhu, Zhang, Ma, Li, Sheng, and Tu^38^ found strong synergism between a tryptophan (VAGW) and a tyrosine (LLLYK)-containing peptide in the ABTS assay, meaning that the particular position of these reactive amino acids in the peptide sequence and the surrounding amino acids greatly impact the type of interactions between peptides. More generally, in their study,^38^ tryptophan-containing peptides (VAGW and APPAMW) displayed a synergistic interaction with a broad variety of antioxidant peptides. This was attributed to the specific location of the tryptophan residue (i.e., the C-terminal position).

When the three faba bean-derived peptides were combined, the interaction was additive, except for the last data point, where moderate synergism was observed. Since the combination of VVIPTEPPHA and TETWNPNHPEL was slightly antagonistic, the combination of VVIPTEPPHA and NYDEGSEPR was synergistic, and the combination of TETWNPNHPEL and NYDEGSEPR was additive, it can be inferred that the combination of the three peptides canceled the synergistic and antagonistic interactions, leading to a global additive effect.

The dose reduction index (DRI) plots were also generated (Figure 2) to evaluate whether the peptide concentration could be reduced when it was used in combination to reach the same level of effect. A DRI of less than one is considered unfavorable, meaning that the peptide dose needs to be increased when used in combination to reach the same level of activity. On the contrary, a DRI above 1 means that dose reduction is favorable and that the peptide concentration can be reduced when used in combination to reach the same level of activity. As log (DRI) is plotted in Figure 2, log (DRI) < 0 is unfavorable, and log (DRI)> 0 is favorable. For all peptide combinations tested, the DRI was favorable, as explained by the additive and synergistic effects. The slightly antagonistic interactions were not sufficient to make the DRI unfavorable. The peptide dose could be reduced by 1.25- upto 18-fold when used in combination to reach the same level of free radical scavenging activity. This finding confirms that the lower EC_50_ measured in the complete 3 kDa permeate of the faba bean flour digestate can be attributed to the additive and synergistic effects of the different peptides. Therefore, the seven potent antioxidant peptides identified in the ABTS assay can be considered important contributors to the overall effect of the antioxidant activity of faba bean flour after gastrointestinal digestion.

For the DPPH assay, the interaction of VIPTEPPHA and TETWNPNHPEL was mostly additive and become slightly antagonistic at low and high levels of free radical scavenging activity. It can be hypothesized that the high proportion of hydrophobic residues in these peptide sequences (Table 1) increases peptide interactions and decreases the availability of reactive residues, tryptophan and histidine to quench the DPPH radical, particularly at high peptide concentrations. Dose reduction was favorable, which again confirms that the lower EC_50_ measured in the complete 3 kDa permeate of faba bean flour digestate can be attributed to the additive effect of the different peptides.

#### 3.1.3 Modulation of the Nuclear Factor Erythroid 2-Related Factor 2-Antioxidant Response Element (Nrf2-ARE) Cell-Signaling Pathway by Faba Bean-Derived Antioxidant Peptides

In addition to direct free radical scavenging, antioxidant peptides can have other modes of action, leading to a protective effect against oxidative stress. One of these process is the modulation of antioxidant cell-signaling pathways. We therefore investigated this potential mode of action of faba bean-derived antioxidant peptides through the Nrf2-ARE live cell assay using a luciferase reporter-gene system. The nuclear factor erythroid 2–related factor 2 (Nrf2) is a transcription factor that induces the expression of several genes that are part of the cell defense system against oxidative stress. When oxidative stress occurs, Nrf2 dissociates from the Kelch-like ECH-associated protein 1 (Keap1) and is translocated from the cytosol to the nucleus, where it binds the antioxidant response element (ARE), an enhancer found in the promoter of several antioxidative enzyme genes, such as the superoxide dismutase (SOD1), the glutathione reductase (GR), and the thioredoxin 1 (Trx1), among others.^42^ Some food-derived antioxidant peptides were shown to activate this pathway by disrupting the interaction between Nrf2 and Keap1^43,44^ and thus, causing the translocation of Nrf2 to the nucleus.

**Figure 3:**
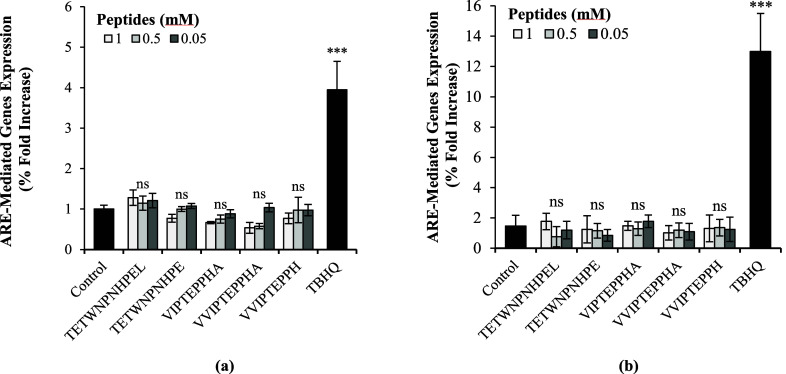
Modulation of the Nrf2-ARE pathway by faba bean-derived antioxidant peptides in: (a) basal conditions; (b) the presence of oxidative stress (H_2_O_2_ 0.25 mM). The different peptide treatments were compared to their respective controls (untreated cells and cells treated with 0.25 mM H_2_O_2_) by one-way Anova and the Dunnett’s posthoc test (***, *p* < 0.001; ns, not significant *p* > 0.05). Tert-butylhydroquinone (TBHQ) was used as a positive control.

We investigated whether the most potent faba bean-derived antioxidant peptides could modulate the Nrf2-ARE signaling antioxidant pathway and thus complement their free radical scavenging properties. Modulation of this cellular antioxidant pathway was tested both in basal conditions and in the presence of oxidative stress (0.25 mM H_2_O_2_). As shown in Figure 3, none of the tested peptides caused a significant increase in ARE-mediated gene expression (*p* > 0.05). This result remains in good agreement with previous studies, where free radical scavenging properties and cell-signaling antioxidant properties were not necessarily correlated.^44^ It is also possible that these peptides failed to induce a cell-signaling effect because of their poor stability and *in vitro* bioavailability. The bioavailability of these peptides will have to be confirmed with subsequent investigations. From these data, we can conclude that the principal mode of action of faba bean-derived antioxidant peptides is through free radical scavenging and not the modulation of the Nrf2 cell signaling pathway or metal ion chelation. Although a dual mechanism of HAT and SET was found for free radical scavenging, the results indicated that the HAT mechanism was favored compared to that of SET. The antioxidant activity of faba bean peptides will need to be tested with *in vivo* assays to confirm the present findings.

### 3.2 Mechanism of ACE Inhibition by Faba Bean-Derived Peptides

#### 3.2.1 ACE Inhibition Activity

**Figure 4:**
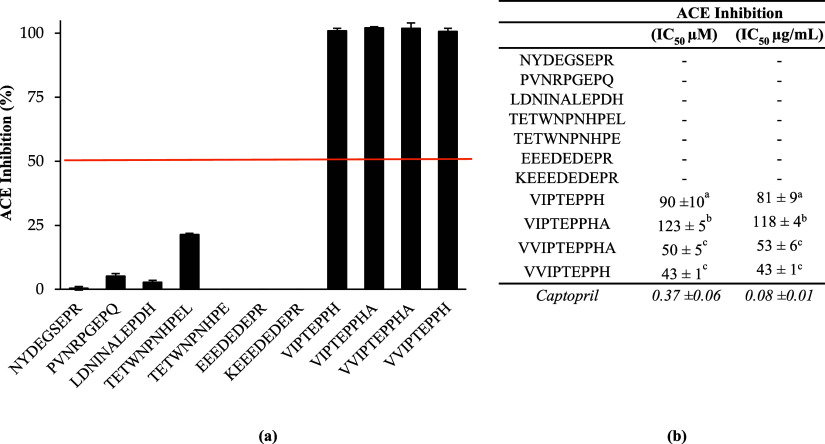
ACE inhibitory activity (mean ± standard deviation) of faba bean-derived peptides after *in vitro* gastrointestinal digestion. Means without a common letter differ (*p* < 0.05), as analyzed by one-way ANOVA and the Tukey’s test. (a) ACE inhibition (%) was determined at 10 mM for all peptides as a first screening. (b) The IC_50_ of the four most potent ACE inhibitory peptides was determined.

The 11 faba bean-derived peptides were screened for ACE inhibition activity. Four peptides, VIPTEPPH, VIPTEPPHA, VVIPTEPPHA, and VVIPTEPPH, demonstrated a strong inhibition activity (Figure 4) where 100% inhibition was obtained at a peptide concentration of 10 mM. In comparison, the nine other peptides had negligible inhibitory activity. The half-maximal ACE inhibitory concentration (IC_50_) of the 4 faba bean peptides ranged from 43 to 123 μM (Figure 4). These values are comparable to other peptides identified in the gastrointestinal digestate of various food products.^45 −47^ Moreover, these four peptides are likely responsible for the ACE inhibitory effect of the complete 3 kDa of faba bean gastrointestinal digestate, since their IC_50_ when expressed in μg/mL (80, 118, 53, and 43 μg/mL, respectively) are significantly lower than the digestate permeate (1348 μg/mL).^16^ Although the inhibitory activity of these peptides is important, it remains 100 to 300 times lower than that of captopril on a molar basis, the latter being a commercialized ACE inhibitor for hypertension treatment.

Despite the similarity in their sequence, the four faba bean peptides were significantly different in their ACE inhibitory potency. For instance, **V**VIPTEPPHA and **V**VIPTEPPH had significantly lower IC_50_ (*p* < 0.05) compared to VIPTEPPHA and VIPTEPPH, respectively, suggesting that the presence of an additional valine residue at the N-terminal extremity of the peptide may play a key role in the ACE inhibitory activity. On the contrary, the presence of an alanine residue at the C-terminal extremity of VIPTEPPH**A** and VVIPTEPPH**A** seems to increase the IC_50_ compared to VIPTEPPH and VVIPTEPPH, respectively.

Noteworthy, the four ACE inhibitor peptides were revealed to be potent antioxidants in the ORAC, ABTS, and/or the DPPH assay, suggesting multifunctionality. This trait is an additional benefit that can serve as a service for the management of hypertension.

#### 3.2.2 ACE Inhibition Pattern of Faba Bean-Derived Peptides

**Figure 5:**
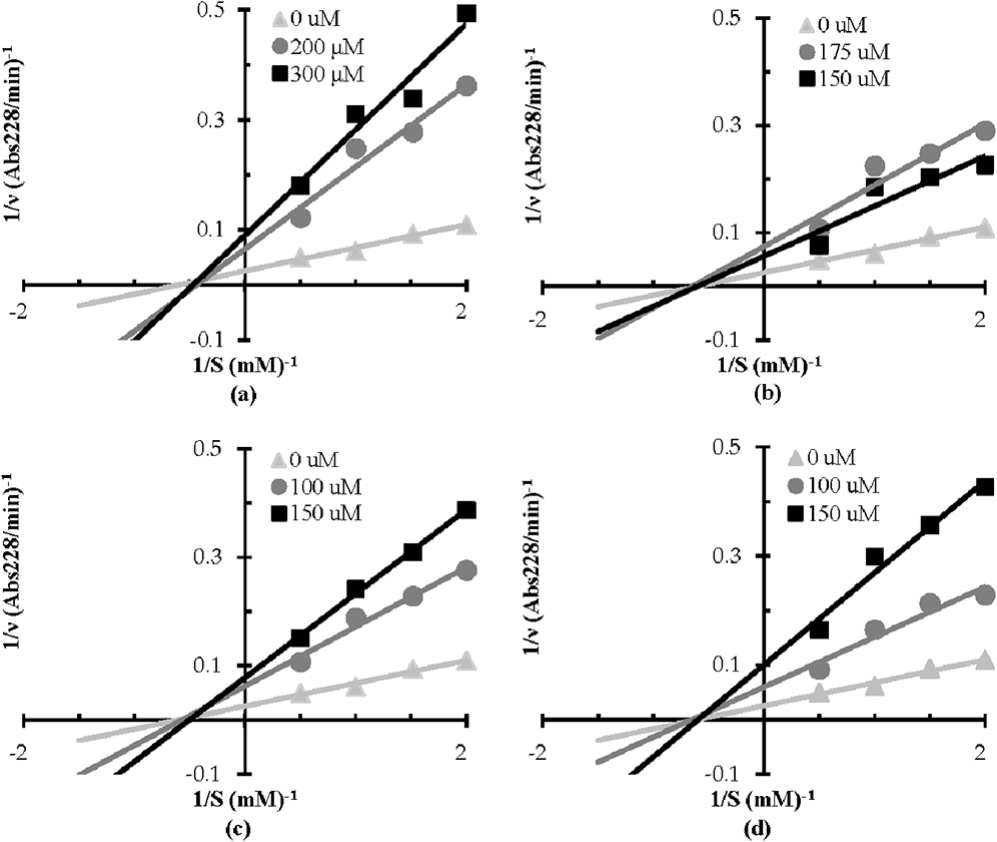
Double reciprocal (Lineweaver–burk) plots of ACE inhibition by faba bean-derived inhibitory peptides. Each point represents the mean of three experiments: (a) VIPTEPPH; (b) VIPTEPPHA; (c) VVIPTEPPHA; and (d) VVIPTEPPH.

The ACE inhibition pattern of VIPTEPPH, VIPTEPPHA, VVIPTEPPHA, and VVIPTEPPH was assessed through kinetic experiments. The initial velocity of reaction was measured at different substrate (0.5–2 mM) and peptide concentrations, and Lineweaver–Burk double reciprocal plots were built to identify the inhibition pattern. As shown in Figure 5, the four peptides exhibited a noncompetitive inhibition pattern. Indeed, in the four cases, the Lineweaver–Burk curves are converging on the *X*-axis, indicating that the apparent *K_m_* is unchanged and the apparent *V*_max_ is decreased with the addition of the inhibitory peptides. This result means that the peptides can bind both the free enzyme and the enzyme–substrate complex with similar affinity. The inhibitor binding site is, therefore, located outside the enzyme active site. The loss of ACE activity in the presence of peptides can, therefore, be explained by conformational changes caused by peptide binding rather than competition for the active site.

The inhibition constant (*K*_*i*_) was calculated from secondary plots for each peptide. The secondary plots were constructed by plotting the Lineweaver–Burk curve slope against the peptide concentration. The *K*_*i*_ value was calculated from the negative intercept on the *X*-axis of the secondary plot. The *K_i_* value was 87 μM for VIPTEPPH, 107 μM for VIPTEPPHA, 45 μM for VVIPTEPPH, and 54 μM for VVIPTEPPHA. These *K*_*i*_ values are nearly identical to the IC_50_, confirming the noncompetitive inhibition pattern. The minor differences in the IC_50_ and *K_i_* values can be explained by experimental imprecision.

#### 3.2.3 Investigation of the Potential Binding Mode Between Faba Bean-Derived Peptides and ACE by Molecular Docking

Molecular docking was used to investigate and compare the potential binding mode of faba bean peptides to ACE. The ACE active site is composed of three substrate binding pockets, namely, S1, S2 and S1.’ S1 is composed of Ala 354, Glu 384, and Tyr 523, S2 is composed of Gln 281, His 353, Lys 511, His 513, and Tyr 520, whereas S1’ is composed of Glu162.^48^ The catalytic mechanism of ACE implies a zinc(II) coordination motif (HEXXH), composed of two histidine (His 383 and His 387) residues and a glutamic acid (Glu411) residue. Commercialized ACE inhibitors, such as captopril and linosipril, are competitive inhibitors^49,50^ of ACE, meaning that they inhibit ACE activity by competing for the active site. Their mechanism of action is well understood and implies direct interaction with the ACE catalytic site composed of a zinc coordination motif in the active site.

For noncompetitive inhibitors, the inhibition mechanism is still not well characterized. Only a few recent studies have attempted to elucidate it.^46,51−54^ Since the kinetic study revealed that the four faba bean peptides (VIPTEPPH, VIPTEPPHA, VVIPTEPPHA, and VVIPTEPPH) act as noncompetitive inhibitors that bind ACE outside the active site, global docking was performed on the whole ACE molecular structure to predict the most probable binding site. HPEPDOCK^26^ was used, which is a docking software that can perform blind flexible protein peptide docking. In this software, peptide flexibility is considered using an ensemble of peptides conformations.^26^

Before performing docking with the four faba bean-derived peptides, the docking procedure was validated with two ACE ligands, the peptide bradykinin-potentiating peptide b (BPPb) (pEGLPPRPKIPP, where pE is a pyroglutamic acid residue) and Angiotensin II (DRVYIHPF), for which cocrystallized structures with ACE are available. The docked peptides were aligned to the peptide structure as found in the ACE-BPPb and ACE-Angiotensin II cocrystallized structures (PDB 4APJ and 4APH, respectively) to calculate the root-mean-square deviation (RMSD) (Table 2). In both cases, the model with the lowest HPEPDOCK docking energy score (model 1) had the lowest RMSD, which was within the generally accepted range of 0–2 Å.^55^ The lowest docking energy score, therefore, leads to the best docking pose in both cases, with the correct orientation. The molecular interactions between the two peptides and ACE were analyzed with LigPlot+ to evaluate whether the principal molecular interactions stabilizing the peptide and ACE complexes were correctly identified. For Angiotensin II, the principal hydrogen bonds with the ACE residue, namely, Gln 281, Tyr 520, Lys 511, His 513, His 383, His 387, and Ala 356, were identified, which is in good agreement with the literature.^56^ For BPPb, the principal hydrogen bonds with Lys 118, Asp 121, Tyr 520, Ser 516, Ser 517, Ala 356, Tyr, 360 and Gln 281 were identified, which again is in good agreement with the literature.^56^ The small variations between the interactions found experimentally by cocrystallization^56^ and with the docking simulation can be explained by a small variation in the docked ligand orientation and software imprecision. Since the RMSD values were in the expected range for the top prediction and the important molecular interactions were successfully identified, the docking protocol was considered reliable and applied to the four faba bean peptides.

**Table 2:** Docking Energy Scores and Root Mean Square Deviation (RMSD) Obtained for BPPb and Angiotensin II in the Docking Validation

	BPPb^a^		angiotensin II	
	docking score	RMSD (Å)	docking score	RMSD (Å)
Model 1	–347.323	0.000	–288.495	0.000
Model 2	–242.122	1.995	–280.668	3.199
Model 3	–239.320	2.004	–271.124	5.026
Model 4	–235.063	1.664	–270.934	4.234
Model 5	–227.442	3.746	–269.242	5.353
Model 6	–226.858	4.147	–266.581	4.295
Model 7	–226.715	3.685	–262.468	3.553
Model 8	–220.964	3.850	–261.550	2.901
Model 9	–219.574	5.991	–255.798	4.485
Model 10	–218.664	3.196	–255.775	3.200

a Bradykinin-potentiating peptide b.

**Figure 6:**
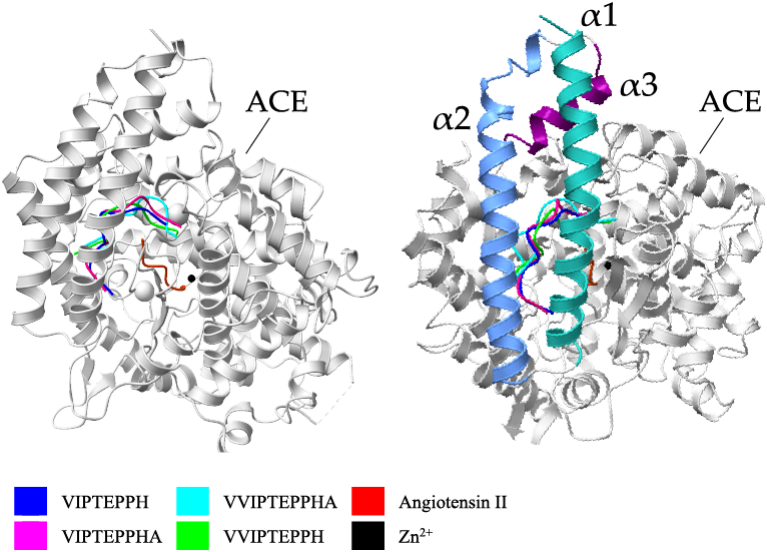
Global views of the best docked poses of faba bean ACE inhibitory peptides with the C-domain of human somatic ACE. Molecular docking simulations were performed using HPEPDOCK^26^ and data visualization was performed using UCSF ChimeraX.^28^.

The docking simulation with the 4 faba bean derived peptides revealed that the most probable binding site of the four faba bean derived peptides is located at the entrance of the active site cavity (Figure 6). The docked peptides were stabilized by hydrogen bonds, hydrophobic interactions, and salt bridges. The ACE active site cavity is ‘protected’ by a ‘lid’ composed of three α helixes of the N-terminal region of ACE α1, α2, and α3, consisting of residues 40–71, 74–107, and 109–120, respectively (Figure 6). These three helixes possess several charged amino acid residues that prevent the entry of large substrates to the ACE active site cavity.^50^ Therefore, the binding of inhibitory peptides in this region is likely to limit the substrate entry and/or product exit from the active site cavity, and thus, decrease the ACE activity.

The four faba bean peptides formed hydrogen bonds with the ACE residue in this region during the docking simulation, namely, Trp 59, Tyr 62, Asn 85, and Thr 92 (Table 3). This binding site is in good agreement with the noncompetitive inhibition mode observed. This mechanism of action was recently proposed for three noncompetitive casein-derived peptides GVSLPEW, GYGGVSLPEW, and VGINYW,^54^ and a Spirulina-derived peptide TMEPGKP.^46^

**Table 3:** Molecular Interactions (Hydrogen Bonds and Salt Bridges) Identified between Faba Bean Peptides (VIPTEPPH, VIPTEPPHA, VVIPTEPPH, and VVIPTEPPHA) and the ACE Residue from Molecular Docking Simulations

ACE (PDB 4APH)^a^	faba bean-derived peptides^b^		
atom name (residue)	atom name (residue)	interaction type	distance (Å)
VIPTEPPH			
NH1 (Arg 124)	O (Pro 3)	hydrogen bond	3.26
NE (Arg 124)	OE2 (Glu 5)	hydrogen bond	3.13
OH (Tyr 62)	O (Pro 3)	hydrogen bond	2.85
VIPTEPPHA			
NH2 (Arg 124)	O (Pro 3)	hydrogen bond	2.28
OH (Tyr 62)	O (Ile 2)	hydrogen bond	3.20
VVIPTEPPHA			
NE2 (His 410)	O (Val 1)	hydrogen bond	2.85
OH (Tyr 360)	N (Val 1)	hydrogen bond	3.18
OH (Tyr 135)	OE1 (Glu 6)	hydrogen bond	2.71
NH2 (Arg 124)	O (Pro 7)	hydrogen bond	3.16
NH2 (Arg 124)	O (Glu 6)	hydrogen bond	2.71
ND2 (Asn 85)	ND1 (His 9)	hydrogen bond	2.63
NE (Arg 124)	OE1 (Glu 6)	salt bridge	3.79
VVIPTEPPH			
NH2 (Arg 522)	O (Val 2)	hydrogen bond	2.81
NE2 (His 410)	N (Val 1)	hydrogen bond	2.94
N Gly 404)	N (Val 1)	Hydrogen bond	3.18
O (Arg 402)	N (Val 1)	Hydrogen bond	2.96
OH (Tyr 394)	N (Val 1)	Hydrogen bond	1.97
O (Asn 136)	ND1 (His 9)	Hydrogen bond	2.56
OG1 (Thr 92)	OE2 (Glu 6)	Hydrogen bond	3.07
OH (Tyr 62)	O (Pro 7)	Hydrogen bond	3.04
NE1 (Trp 59)	O61 (Thr 5)	Hydrogen bond	2.52

a Angiotensin-converting enzyme (ACE) in complex with angiotensin-II.

Hydrogen bonds are important molecular interactions that stabilize molecular complexes. During the docking simulations, there were significant differences in the number of hydrogen bonds formed with ACE residue, which can explain the different inhibitory potency among the four faba bean peptides (Table 3). The peptides VVIPTEPPH and VVIPTEPPHA were stabilized by a higher number of hydrogen bonds (9 and 7, respectively) compared to VIPTEPPH and VIPTEPPHA (3 and 2, respectively), which is in good agreement with their inhibitory activity potencies. Moreover, VVIPTEPPH and VVIPTEPPHA formed hydrogen bonds with ACE residues that are closer to the active site pockets, namely, His 410, Arg 522, Gly 404, Arg 402, and Tyr 394, which again could explain their higher activities. More specifically, the first valine residue in VVIPTEPPH and VVIPTEPPHA formed 4 and 2 hydrogen bonds, respectively, with the ACE residue, confirming the importance of this residue in the stabilization of the inhibitory peptide and ACE complexes. The valine at the N-terminal extremity of VVIPTEPPH and VVIPTEPPHA formed a hydrogen bond with His 410, which is right next to Glu 411, an important residue of the ACE catalytic center. Therefore, the results of the docking simulation are in good agreement with the noncompetitive inhibition pattern of the four peptides and their inhibitory activity potency.

This study reported, for the first time, a comprehensive investigation of the mechanism of action of faba bean-derived bioactive peptides after *in vitro* gastrointestinal digestion. The mechanism of action of 7 novel bioactive peptides derived from faba bean flour gastrointestinal digestate was ascertained. NYDEGSEPR, TETWNPNHPEL, TETWNPNHPE, VIPTEPPH, VIPTEPPHA, VVIPTEPPHA, and VVIPTEPPH were revealed to be potent antioxidant peptides, through free radical scavenging, principally through a HAT-based mechanism. Combinations of faba bean peptides lead mostly to an additive and/or synergistic antioxidant effect, which indicates the importance of consuming faba bean proteins as a whole ingredient. Four peptides, namely VIPTEPPH, VIPTEPPHA, VVIPTEPPHA, and VVIPTEPPH, were also potent ACE inhibitory peptides, making them multifunctional, which is of great interest in the management of noncommunicable diseases. The four peptides are noncompetitive inhibitors of ACE, and their most probable binding sites are located near the entrance of the active site cavity. From these results, it can be concluded that the antioxidant and ACE inhibitory activities of faba bean flour after *in vitro* gastrointestinal digestion can be associated with the release of bioactive peptides with synergistic and multifunctional activities. Future research will be needed to investigate the bioavailability of these peptides to confirm their bioactive potential. *In vivo* assays will also need to be performed to confirm the bioactive properties of faba bean-derived peptides after gastrointestinal digestion.
